# Traditional Chinese Medicine Monomers: Novel Strategy for Endogenous Neural Stem Cells Activation After Stroke

**DOI:** 10.3389/fncel.2021.628115

**Published:** 2021-02-26

**Authors:** Ju Wang, Jun Hu, Xuezhu Chen, Xuejiao Lei, Hua Feng, Feng Wan, Liang Tan

**Affiliations:** ^1^Department of Neurosurgery and Key Laboratory of Neurotrauma, Southwest Hospital, Third Military Medical University (Army Military Medical University), Chongqing, China; ^2^Department of Neurology, Southwest Hospital, Third Military Medical University (Army Military Medical University), Chongqing, China; ^3^Department of Electrical and Computer Engineering, Faculty of Science and Technology, University of Macau, Macau, China

**Keywords:** stroke, neural stem cells, traditional Chinese medicine monomers, neuroregeneration, central nervous system

## Abstract

Stem cell therapy, which has become a potential regenerative medical treatment and a promising approach for treating brain injuries induced by different types of cerebrovascular disease, has various application methods. Activation of endogenous neural stem cells (NSCs) can enable infarcted neuron replacement and promote neural networks’ regeneration without the technical and ethical issues associated with the transplantation of exogenous stem cells. Thus, NSC activation can be a feasible strategy to treat central nervous system (CNS) injury. The potential molecular mechanisms of drug therapy for the activation of endogenous NSCs have gradually been revealed by researchers. Traditional Chinese medicine monomers (TCMs) are active components extracted from Chinese herbs, and some of them have demonstrated the potential to activate proliferation and neurogenesis of NSCs in CNS diseases. Ginsenoside Rg1, astragaloside IV (AST), icariin (ICA), salvianolic acid B (Sal B), resveratrol (RES), curcumin, artesunate (ART), and ginkgolide B (GB) have positive effects on NSCs *via* different signaling pathways and molecules, such as the Wingless/integrated/β-catenin (Wnt/β-catenin) signaling pathway, the sonic hedgehog (Shh) signaling pathway, brain-derived neurotrophic factor (BDNF), nuclear factor erythroid 2-related factor 2 (Nrf2), and heme oxygenase 1 (HO-1). This article may provide further motivation for researchers to take advantage of TCMs in studies on CNS injury and stem cell therapy.

## Introduction

Almost 15 million people are affected by stroke every year, and because most are left disabled, stroke is a leading cause of disease burden worldwide (Johnston et al., 2009). Stroke, which is a sudden interruption of the blood supply to the brain, leads to neurological deficits, and the most effective treatments can only be applied in fewer than 20% of patients (Ojaghihaghighi et al., 2017). A series of pathophysiological changes appear after blood supply interruption, including local oxygen free radical and reactive oxygen species generation, cerebral edema, neuroinflammation, local inflammatory cell infiltration, and blood-brain barrier (BBB) destruction, resulting in irreparable neural disruption (Khoshnam et al., 2017; Datta et al., 2020). Neural stem cells (NSCs) can be activated, differentiate into different cell types, and migrate to ischemic lesions to promote postinjury repair through nutritional support, inflammatory response regulation, and functional restoration, indicating that NSCs are prime candidates for use in regenerative medicine (Grochowski et al., 2018). In the basic and clinical stroke research fields, stem cell therapy is considered to be a promising regenerative medical treatment and a promising approach for stroke (Sarmah et al., 2018). Among all the different methods of stem cell therapy, transplantation of exogenous stem cells and activation of endogenous or dormant stem cells are the primary safe and effective candidate strategies for stroke treatment (Yasuhara et al., 2020).

A variety of transplantable cell types, including embryonic stem cells (ESCs), adult tissue-derived stem cells, gene-edited stem cells, and induced pluripotent stem cells (iPSCs), have been used to generate both protective and regenerative effects in the stroke brain in laboratory studies. Tornero et al. (2017) transplanted iPSCs into stroke-injured adult rat cortices and observed improvements in neurological deficits and enhancement of the morphological development of both afferent and efferent nerves as well as their functional connections with host cortical neurons (Oki et al., 2012). Furthermore, grafted neurons can integrate into adult host neural networks and even into the brain’s neural circuitry (Grønning Hansen et al., 2020; Palma-Tortosa et al., 2020). Researchers have adapted intravenous (IV) administration of autologous mesenchymal stem cells (MSCs), which has been studied extensively in animal models, for use in patients with ischemic stroke, and positive clinical outcomes have been achieved after stroke onset (Díez-Tejedor et al., 2014). However, successful transplantation of exogenous stem cells is still hindered by several technical and logistical problems. The specific cell type used is the principal concern due to ethical issues, and researchers must also consider the source as well as the location, dosage, route, and timing of administration (Fernández-Susavila et al., 2019; Kawabori et al., 2020). A series of conditions following a stroke, including neuroinflammation, and immune response activation not only seriously affect the survival of grafted NSCs but also facilitate their differentiation into glial cells; the potential tumorigenicity of transplanted cells is also a concern (Ideguchi et al., 2008; Datta et al., 2020). Microglia in the proinflammatory state may restrain phagocytes from clearing toxic debris and secreting tissue-repairing neurotrophic factors while contributing to neuronal cell death or further BBB disruption or impairing axonal regeneration (Lyu et al., 2020). Besides, it would be costly and labor-intensive to generate autologous iPSCs for personalized medicine in practice, and such a strategy would introduce the possibility of genetic defects that would reduce the therapeutic efficacy (Bang and Kim, 2019).

Therefore, activation of endogenous NSCs, with benefits including improvements in proliferation, directional migration, and differentiation, could be a more practical therapeutic approach to decrease stroke damage without the above disadvantages. The therapeutic effects of this approach can be achieved through nutritional support, regulation of the inflammatory response, directional replacement of neurons, reconstruction of neural circuits, restoration of neural function, and paracrine signaling involving nerve growth factor (NGF; Bain et al., 2013; Yu et al., 2016). Endogenous NSCs, which are abundant in the subgranular zone of the hippocampus and the subventricular zone of the germinal region of the brain, can differentiate into various types of cells to promote structural and functional repair of the brain and spinal cord tissue (Daynac et al., 2016; Song et al., 2018). However, the disastrous changes in the cellular microenvironment after stroke due to BBB damage, excitatory toxicity and neuroinflammation impact the survival, neurogenesis, and differentiation of NSCs (Zhong et al., 2021). The endogenous NSCs tend to differentiate into gliocytes rather than neurons, and the majority of them fail to reach the lesioned cortex. Therefore, developing an efficient and safe strategy to activate the transformation of endogenous NSCs into newborn neurons seems to be significant (Parent et al., 2002; Kreuzberg et al., 2010).

The use of traditional Chinese medicines, whether in the form of herbs, formulas, or monomers, to prevent and treat diseases of the central nervous system (CNS) has been a popular research topic for decades and has shown significant curative effects in many cases. A recent meta-analysis analyzed 67 randomized controlled trials and indicated that treatment with traditional Chinese medicines can enhance the clinical rehabilitation of stroke patients and significantly improve the recovery of neurological function to improve patient quality of life (Zhang et al., 2020). The relationship between traditional Chinese medicine treatments and stem cell therapy after stroke has also been investigated, and published studies have shown that these treatments benefit neural regeneration by promoting the proliferation and differentiation of NSCs (Li et al., 2015; Gao et al., 2017). Traditional Chinese medicine monomers (TCMs) are essential drugs that promote activation of endogenous NSCs after stroke. An increasing number of studies have explored the effects of TCMs on stroke. In this review article, we summarized the efficacy and mechanisms of TCMs that have been reported to activate NSCs in stroke (Table 1).

**Table 1:** Effect of different traditional Chinese medicine monomers (TCM) on neural stem cells (NSCs) after stroke *in vitro* and *in vivo*.

Types	Sources	Components	Dose	Model	Objects	Pathways	Mechanisms	Stroke outcomes	References
Glycosides	*Panax notoginseng* (Burk) F. H. Chen	Ginsenoside Rg1	0.32 μg/ml	OGD	*In vitro*	None	Proliferation ↑ Differentiation ↑	NA	Gao et al. (2017)
			10, 20 μM/L	OGD	*In vitro*	Caspase 3 and Bax ↓ Bcl-2 ↑ P38 and JNK2 phosphorylation ↓	Apoptosis ↓ Oxidative stress ↓	NA	Li et al. (2017)
	*Astragalus membranaceous* (Fisch.) Bge. var. *mongholicus* (Bge.) Hsiao	Astragaloside IV	40 mg/4 ml once daily for 14 days	MCAO	Rat	BDNF-TrkB signaling pathway ↑	Proliferation ↑ Migration↑ Differentiation ↑ Maturation↑	Neurological behavioral deficits ↓ Infarction volume ↓	Ni et al. (2020)
			200 mg/kg *in vivo* 20 μM, *in vitro*	MCAO/Primary cell	*In vitro*/ Mouse	PI3K/Akt pathway↑ Wnt/β-catenin pathway ↑ IL-7 ↓	Apoptosis ↓ Proliferation ↑ Differentiation ↑	Anxiety-like behavior ↓	Sun et al. (2020)
Polyphenols	*Epimedium brevicornu* Maxim	Icariin	100 nM/L	None	*In vitro*	ERK	Proliferation ↑	NA	Huang et al. (2014)
			50, 100 μM/L	None	*In vitro*	Cyclin D1 and p21 ↑	Proliferation ↑	NA	Fu et al. (2018)
	*Salvia miltiorrhiza* Bunge (Lamiaceae)	Salvianolic acid B	50 μM/L once daily for 2 weeks	None	*In vitro*	PI3K/Akt signal pathway	Proliferation ↑	NA	Zhuang et al. (2012)
	Extract of *Veratrum grandiflorum*	Resveratrol	5 μmol/L	OGD	*In vitro*	SOD, Nrf2, HO-1, and NQO1↑	Apoptosis ↓ Oxidative stress ↓ Proliferation ↑	NA	Shen et al. (2016)
			5 μmol/L	OGD	*In vitro*	Shh signaling pathway ↑	Proliferation	NA	Cheng et al. (2015)
	Extract of *Curcuma longa* L	Curcumin	20 mg/kg, *in vivo* 0.5 μM, *in vitro*	None	*In vitro*/Rat	Wnt/β-Catenin Signaling pathway ↑	Proliferation ↑ Differentiation ↑	NA	Tiwari et al. (2014)
			1, 5 mg/ml	ICH	Rat	None	Oxidative stress ↓	Neurological behavioral deficits ↓ Hematoma size ↓	Marques et al. (2020)
Terpenoids	Extrcat of *Artemesia annua*	Artesunate	150 mg/kg, *in vivo* 0.8 μmol/L, *In vitro*	OGD/MCAO	*In vitro*/ Mouse	PI3K/Akt/FOXO-3a/p27Kip1 signaling pathway	Proliferation ↑	Neurological behavioral deficits ↓ Infarction volume ↓	Zhang et al. (2020)
	*Ginkgo biloba* L	Ginkgolide B	20 mg/kg, *in vivo* 40 and 60 mg/L, *in vitro*	MCAO	Rat	BDNF↑	Proliferation ↑ Differentiation ↑	Neurological behavioral deficits ↓	Zheng et al. (2018)

↑, up-regulation; ↓, down-regulation.

## Glycosides

### Ginsenoside Rg1

Ginsenoside Rg1 is the most abundant component isolated from Panax notoginseng saponins (PNS), the major bioactive components extracted from the root of *Panax notoginseng* (Burk) F. H. Chen, can significantly reduce infarction volume and alleviate neurological deficits caused by cerebral ischemia/reperfusion (I/R; Zeng et al., 2014). Yang et al. (2020) speculated that the mechanisms of their neuroprotective effects could be related to anti-oxidative stress effects, anti-inflammatory and anti-apoptotic effects, promotion of BBB repair, and prevention of calcium overload. Furthermore, PNS are the primary ingredients of PNS tablet, Xuesaitong Soft Capsule, Xuesaitong Injection, and Xueshuantong Injection, all of which have been used in the treatment of acute ischemic stroke for many years in China (Yao et al., 2011; Yang et al., 2013; Li et al., 2018). Clinical research has suggested that Xuesaitong Soft Capsule relieves unstable angina with few side-effects (Yang et al., 2013). Li et al. (2012) included 30,884 cases in a new systematic post-marketing safety surveillance study to re-evaluate Xuesaitong Injection and found that it is safe for clinical use. Also, this medicine upregulates the expression of brain-derived neurotrophic factor (BDNF), a critical factor that promotes the repair and reconstruction of neurons associated with learning and memory and inhibits Tau protein phosphorylation in Alzheimer’s disease (AD; Li et al., 2012). BDNF and its receptor tyrosine kinase receptor B (Trk B), have been proven to be closely related to the regulation of hippocampal neuron survival, nerve repair, and regeneration. Some studies have drawn attention to the association between serum BDNF and functional outcomes after stroke, but there seems to be little related evidence (Luo et al., 2019).

The effects of ginsenoside Rg1 in stem cells have been demonstrated. Ginsenoside Rg1 can dose-dependently promote the expression of neural cell adhesion molecule (NCAM) and synapsin-1 (SYN-1) to improve the cell proliferation and neural phenotype differentiation of both human and mouse adipose-derived stem cells (Xu et al., 2014; Dong et al., 2017). Rg1 has positive effects on the proliferation and differentiation of endogenous NSCs in AD rats and promotes the functional expression and maturation of human NSCs (Jiang et al., 2012; Zhao et al., 2018). Extracellular regulated kinases 1 and 2 (ERK1 and ERK2), which mediate neuroinflammation, may be vital molecular targets involved in Rg1-induced NSCs differentiation, and the effects of Rg1 on NSCs proliferation are most closely related to mechanisms involving the Hes1, cyclic adenosine monophosphate (CAMP)-protein kinase A (PKA) and phosphatidylinositol 3 kinase (PI3K)-AKT signal transduction pathways (Zhuang et al., 2009; Li et al., 2012; Zhao et al., 2012). As an effector of Notch signaling, which can improve the maintenance of NSCs during development, Hes 1 plays an important role in NSCs proliferation besides its role in astrogenesis (Kageyama et al., 2020). Metabolic abnormalities increase the risk of stroke, and reduce recanalization rates and increase the risk of hemorrhage, making tissue-type plasminogen activator (tPA) treatment potentially hazardous for stroke patients with diabetes mellitus or poststroke hyperglycemia (Jiang et al., 2020; Zhou et al., 2020). In mice subjected to middle cerebral artery occlusion (MCAO), administration of ginsenoside Rg1 was found to attenuate energy undersupply and amino acid and lipid metabolic disturbances and thereby to ameliorate neurological deficits and cerebral infarcts; however, slight differences were found between ginsenoside Rg1 treatment alone and combined NSCs transplantation and ginsenoside Rg1 treatment (Gao et al., 2020). Grafted ginsenoside Rg1-induced NSCs improved the learning and memory behavior in rat neonatal hypoxic-ischemic encephalopathy models. However, the isolated effects of ginsenoside Rg 1 on activating NSCs after stroke were unclear (Li et al., 2015). Increasing evidence has indicated the positive impact of Rg1 on NSC proliferation after oxygen-glucose deprivation (OGD), and glial-like directed differentiation is another effect of Rg1 on NSCs (Gao et al., 2017). Rg1 (10–20 μM) protects NSCs from OGD-induced cell death *via* apoptotic signaling and inhibits p38 and c-Jun N-terminal kinase2 (JNK2) phosphorylation to protect NSCs against oxidative stress (Li et al., 2017). The ginsenoside metabolite 20(S)-protopanaxadiol promotes the transition of NSCs from a state of proliferation to differentiation *via* induction of autophagy and cell cycle arrest, indicating that the process of ginsenoside metabolism may be directly involved in the activation of endogenous NSCs after stroke (Chen et al., 2020).

### Astragaloside IV

Astragaloside IV (AST) is purified from the root of *Astragalus membranaceus* (Fisch.) Bge. var. *mongholicus* (Bge.) Hsiao and has been widely used in China for the treatment of hepatic, cardiovascular, and renal disorders (Fu et al., 2014). A systematic review performed to elucidate the relationships between AST and neurological disorders has revealed that the positive effects of AST on neurological disorders are due mainly to its anti-edema effect, which reduces lymphocyte infiltration and the numbers of dopaminergic neurons (Costa et al., 2019). Wang et al. (2017) searched eight databases to assess the function and possible mechanisms of AST in experimental stroke. Their investigation demonstrated that AST improves neurological deficits and reduces infarct volumes and BBB permeability and its antioxidant, anti-inflammatory and antiapoptotic properties provided neuroprotection during cerebral I/R injury (Wang et al., 2017).

The possible mechanisms of AST related to the proliferation of NSCs have been gradually revealed. Low-dose (10^−6^/10^−7^ M) AST increases NSCs proliferation, high-dose (10^−5^ M) AST has no effects on cells, and low-dose of AST promotes NSCs differentiation partly through the Notch signaling pathway *in vitro* (Haiyan et al., 2016). Observed increases in the numbers of BrdU-positive and DCX-positive cells have proven that AST promotes adult neurogenesis in the mouse hippocampal dentate gyrus by regulating signal transduction through the chemokine (chemotactic cytokine)-family protein ligands CXCL1/CXCR *in vivo* (Huang et al., 2018). Chemokines and their receptors are widely expressed in the CNS, and CXCL 2 can enhance the viability of hippocampal neurons. Gao et al. (2018) confirmed that AST can induce the differentiation of NSCs into midbrain dopamine (DA) neurons, possibly through upregulation of sonic hedgehog (Shh), orphan nuclear hormone (Nurr1), and pituitary homeobox 3 (Ptx3), in Parkinson’s disease (PD). Wang et al. (2009) proposed that upregulation of the expression of NGF in the adult hippocampus, which is crucial for the survival and maintenance of neurons after stroke, is related to AST-induced proliferation and the neuronal differentiation of endogenous NSCs after transient forebrain ischemia. In a study conducted by Ni et al. (2020) after MCAO, Sprague–Dawley rats were intragastrically administered AST (40 mg/4 ml/kg, once daily) for 14 days to investigate the effect of AST after stroke. The results showed that AST treatment reduced the infarct volume, improved behavior, increased body weight, and promoted proliferation, migration, differentiation, and maturation of NSCs in the hippocampus by enhancing the BDNF-TrkB signaling pathway (Ni et al., 2020). Sun et al. (2020) discovered that interleukin (IL)-17, a proinflammatory cytokine, is a key effector of AST that regulates NSCs apoptosis and proliferation by upregulating the Wnt/β-catenin and Akt/GSK-3β pathways after the administration of 200 mg/kg AST *via* the tail vein for three consecutive days in MCAO model.

## Polyphenols

### Flavonoids

*Epimedium brevicornu* Maxim, a traditional Chinese herb, is usually used to treat impotence, numbness, and osteoporosis (Pei et al., 2008). Icariin (ICA), the principal representative flavonoid of this herb, has shown potential preventive and therapeutic effects in nervous system diseases. ICA treatment accelerates locomotor function recovery, decreases the expression of inflammatory molecules, and enhances the expression of anti-inflammatory proteins after spinal cord injury (SCI), which proves that its protective effects may be induced by its antiapoptotic, antioxidant, and anti-inflammatory bioactivities (Jia et al., 2019). The results of a Morris water-maze test have indicated that ICA attenuates cognitive functional deficits in aging rats (Wu et al., 2012). Xiong et al. (2016) found that pretreatment with ICA reduces neurological deficit scores and infarct volumes through inhibition of inflammatory responses mediated by nuclear factor κB (NF-κB) and the peroxisome proliferator-activated receptor (PPAR) isotypes PPARα and PPARγ. Another PPAR isotype plays essential roles in cell proliferation and differentiation, lipid and glucose metabolism regulation, and inflammatory and oxidative responses. The anti-autophagic and anti-apoptotic properties of ICA protect mouse MSCs from OGD damage (Liu et al., 2020).

After the isolation and characterization of NSCs derived from 16 to 20-week-old human fetuses, Yang et al. (2016) found that low doses (0.1 and 1 μM) of ICA had a minimal effect on the proliferation of NSCs; conversely, a high dose (10 μM) markedly promoted proliferation and regulated related gene expression in human NSCs *in vitro*. Huang et al. (2014) found that treatment with 100 nmol/L ICA promoted the proliferation and neurosphere formation of mouse NSCs induced by ERK but had no effect on the differentiation of NSCs. Also, ICA can cause upregulation of the cell cycle genes cyclin D1 and p21 to affect the growth and proliferation of NSCs (Fu et al., 2018). ICA is also capable of activating quiescent NSCs in the hippocampi of aging (18- to 21-month-old) rats (Wu et al., 2012; Liu et al., 2020). The results of a study by Liu et al. (2018) show that after transient MCAO, combined ICA and MSCs treatment decreases the brain infarct volume, improved neurological motor deficits, and increases the expression of VEGF and BDNF to maximum levels in the hippocampus and frontal cortex through the phosphatidylinositide 3 kinase (PI3K)/ERK1/2 pathway. VEGF can promote endothelial cell regeneration and new blood vessel formation to protect learning and memory. ICA promotes the proliferation and differentiation of β-amyloid protein (Aβ_25–35_)-treated hippocampal NSCs *via* the BDNF-TrkB-ERK/Akt signaling pathway, indicating that ICA has a positive effect on NSCs in CNS diseases (Quan et al., 2020). A growing number of researchers have paid attention to the applications of ICA in stem cell therapy because of the beneficial effects of this compound.

### Phenolic Acids

Salvianolic acid B (Sal B) is a water-soluble product of *Salvia miltiorrhiza* Bunge (Lamiaceae) known as a treatment for cardiovascular and cerebrovascular diseases (Zhou et al., 2005). It is an ingredient in many formulas for stroke, such as Salvianolate Lyophilized Injection, Naoxintong, and *Salviae miltiorrliza*-borneol Jun-Shi coupled-herbs (Chen et al., 2016; Xue et al., 2016; Duan et al., 2019). Sal B reduces the memory impairments induced by cholinergic dysfunction and Aβ_25–35_, and the protective mechanisms may be associated with its antioxidant and anti-inflammatory properties (Kim et al., 2011). In a microglia-neuron coculture system, Sal B was found to inhibit activation of microglia, and low production of nitric oxide (NO), tumor necrosis factor-α (TNF-α), IL-1β, and reactive oxygen species in hippocampal neurons indicated that it had a neuroprotective effect (Wang et al., 2010). Sal B treatment has been proven to be effective in stroke and to attenuate brain injury by reducing infarct volume and brain edema and increasing neurological scores; those effects are accompanied by inhibition of apoptosis and inflammation *via* activation of silent information regulator 1 (SIRT1) signaling, and has been proven to be effective in stroke (Lv et al., 2015). Delayed postischemic treatment with Sal B improves cognitive impairment after stroke in rats (Zhuang et al., 2012).

Multiple studies have focused on the effect of Sal B in stem cells. Sal B (1 μM) induces human ESCs to differentiate into hepatocytes, and the activation of the Wnt/β-catenin signaling pathway and the inhibition of the Notch pathway are involved in this process (Chen et al., 2018). Guo et al. (2010) found that Sal B treatment results in a substantial increase in stem cell proliferation, differentiation into neurons, and self-renewal *in vitro*. A study by Zhuang et al. (2012) reached the same conclusion and showed that the PI3K/Akt signaling pathway might be induced. A series of studies by Shu et al. (2015); Shu et al. (2018) have demonstrated that Sal B can potently not only potently promote iPSC proliferation and differentiation into neurons but also markedly attenuate the apoptotic ratio of iPSC-derived NSCs, possibly through the PI3K/AKT/glycogen synthase kinase 3β/β-catenin (PI3K/AKT/GSK3β/β-catenin) pathway. Thus, Sal B may be useful as an alternative treatment for the activation of endogenous NSCs. Yan et al. (2020) found that combined application of Sal B and transplantation of MSCs is more effective in degenerated intervertebral disc repair than Sal B treatment or MSCs transplantation alone. However, as mentioned before, the effect is dose-dependent, and the cost is high.

### Others

#### Resveratrol

Since it was first isolated from *Veratrum grandiflorum* in 1940, resveratrol (RES or 3,5,4′-trihydroxystilbene) has been reported to be an effective extract of many foods and Chinese herbs (Gambini et al., 2015). RES, a nonflavonoid polyphenol phytoalexin, has been used in medicinal preparations, such as *munakka* or *darakchasava*, for more than 2,000 years (Paul et al., 1999). It has been reported that RES reverses multidrug resistance in cancer cells and sensitizes cancer cells to standard chemotherapeutic agents when combined with clinically used drugs (Ko et al., 2017). A randomized, double-blind placebo-controlled phase II trial of RES in patients with mild to moderate AD including a total of 104 participants clearly showed that RES was detectable at a safe and well-tolerated concentration in the cerebrospinal fluid, indicating the feasibility of using RES to treat neurodegenerative diseases. Furthermore, RES modulates the CNS immune response by facilitating the activation of microglia/macrophages (Sawda et al., 2017). Besides, RES has been shown to have a positive effect against stroke by upregulating the antioxidative stress molecule Nrf2, proving the antioxidative activity of this compound (Shen et al., 2016).

Consistent with the multiple effects of RES, researchers have found that different RES working concentrations and treatment times can lead to the opposite effects in pluripotent stem cells (PSCs). Li et al. (2017) found that 50 or 500 nM RES maintained the pluripotent state of mouse ESCs after cell differentiation and that the kinase/signal transducers and activators of transcription 3 (JAK/STAT3) signaling pathway were activated, whereas the mammalian target of rapamycin (mTOR) signaling pathway was suppressed, to reinforce the self-renewal of mouse ESCs. However, Park et al. (2012) reported that when 20 and 50 μM RES were added to the stem cell culture medium, the proliferation of neural stem progenitor cells (NSPCs) was inhibited, and the proliferation of self-renewing cells in the hippocampus was reduced. Treatment with 10 μM RES delays entry into the S phase of the cell cycle and affects the mitochondrial metabolism of mouse ESCs, and adenosine monophosphate-activated protein kinase, and Unc-51-like autophagy activating kinase 1 (AMPK/ULk1)-dependent autophagy enhance the pluripotency of ESCs (Suvorova et al., 2019). RES (10 mM) maintains the stemness characteristics of human ESCs and promotes their proliferation through SIRT1/ERK signaling but did not influence the apoptotic rate of the cells (Safaeinejad et al., 2017). RES also promotes the long-term survival and neuronal differentiation of human NPCs in the rat brain (Yao et al., 2020). In one study, various concentrations of RES (1–100 μmol/L) were added to the culture medium, and the finding that there were more BrdU-positive cells in the 5 μmol/L group than in the OGD group demonstrated that RES increased the proliferation of NSCs after OGD.

Further research by Cheng et al. (2015) has suggested that the Shh signaling pathway mediates this renewal process. An experiment with a similar experimental design and dose was performed by Shen et al. (2016) but the researchers selected antioxidative stress-related signaling as an interventional target. The results proved that RES protected NSCs from apoptosis, increased the proportions of NSCs in the S and G2/M phases, and significantly upregulated the expression levels of Nrf2, heme oxygenase 1 (HO-1), and NAD(P)H: quinone oxidoreductase 1 (NQO1) after ODG, indicating that RES is capable of positively regulating NSCs in stroke (Shen et al., 2016).

#### Curcumin

Curcumin is the most abundant phytoconstituent among the curcuminoids and is extracted from the powdered rhizomes of *Curcuma longa* L (Priyadarsini, 2014). Curcumin is well known for its anti-inflammatory, anti-mutagenic, anti-carcinogenic, antimicrobial, antifibrotic, and antioxidant actions and has several effects on the CNS. The antitumor effect of curcumin is due to its inhibition of the growth, cell cycle progression, migration, and invasion of glioma cells, and most of these effects are remarkably dependent on mTOR-dependent ATG induction (Ryskalin et al., 2020). Curcumin has been reported to inhibit most activities of Aβ, including aggregation and Aβ-induced inflammation, *in vitro*, and oral administration of curcumin can improve behavioral impairments, inhibit Aβ oligomerization and deposition, and inhibit tau phosphorylation in the brains of AD animal models (Hamaguchi et al., 2010). Curcumin exerts protective effects against I/R injury by inducing the oxidative stress response, leukocyte infiltration, complement activation, and mitochondria-mediated mechanisms and protecting the BBB against disruption (Bavarsad et al., 2019).

It has been proven that neurogenesis in the context of stem cell therapy is another critical area in which curcumin functions. Kim et al. (2008) found that administration of 0.1 and 0.5 μM curcumin *via* direct dilution into the culture medium of hippocampal NSCs promoted NSC proliferation, whereas 10 μM curcumin had the opposite effect, indicating that the dose of curcumin is a crucial factor affecting the proliferation of NSCs. Also, Son et al. (2014) cultured spinal cord NSPCs with curcumin and reached the same conclusions regarding the dose of curcumin and the MAPK signaling pathway-mediated proliferation of NSCs. However, curcumin has some shortcomings, such as low absorption and metabolism, low water solubility, and rapid excretion from the blood circulation. Tiwari et al. (2014) applied curcumin-encapsulated PLGA nanoparticles (Cur-PLGA-NPs) to prove the positive influence of curcumin on adult neurogenesis. The results showed that even as little as 0.001 μM bulk curcumin promoted NSCs proliferation *in vitro* and that activation of the canonical Wnt/β-catenin pathway was involved in this process (Tiwari et al., 2014). Nanoemulsified curcumin could improve neurological behavioral deficits, decrease the size of hematoma and elicit antioxidant responses without exhibiting cytotoxicity in the liver and kidney after stroke (Marques et al., 2020). The water-insolubility and low bioavailability of curcumin may impose restrictions on its passage through the BBB, but biocompatible formulations of organic substances and novel metal and oxide nanoparticles can be developed to increase its ability to pass through the BBB. Curcumin has the potential to play a more practical role in stem cell therapy after stroke due to its effects on other nervous system diseases.

## Terpenoids

### Artesunate

Artemisinin is a sesquiterpene lactone peroxide isolated from the leaves of the shrub *Artemesia annua* that is usually applied as an anti-malaria drug (Mohammadi et al., 2020). Artesunate (ART), also known as dihydroartemisinin-12-α-succinate, is a semisynthetic antimalarial compound derived from artemisinin that has high efficacy, solubility, and bioavailability and can be administered in many ways, including *via* the IV, intramuscular (IM), oral and rectal routes (Morris et al., 2011). Because of the superior qualities of ART, many of its effects have been investigated. ART can significantly induce cancer cell apoptosis, inhibit glioma cell growth and migration, and influence glioma cell metabolism (Wei et al., 2020). Gugliandolo et al. (2018) proved that ART can ameliorate traumatic brain injury (TBI)-induced lesions through its anti-inflammatory activity, and its protective effects are mediated through modulation of neurotrophic factors that play crucial roles in neuronal survival. Treatment with 200 mg/kg ART has been found to improve the modified Garcia score and decreased the brain water content in an animal model of subarachnoid hemorrhage (SAH). Moreover, ART treatment protects the BBB through activation of sphingosine-1-phosphate receptor-1 (S1P1), enhancement of PI3K activation, and stabilization of β-catenin in SAH mice (Zuo et al., 2017). *Via* immunofluorescence staining, Zhang et al. (2020) found remarkably enhanced numbers of cells in the G2/S phase and proliferation among NSPCs treated with 0.8 μmol/L ART. Also, PI3K/Akt/FOXO-3a/p27/Kip1 signaling is involved in the effects of ART after OGD *in vitro* and MCAO *in vivo*, and motor function recovery data indicate that ART may be a potential therapeutic agent for ischemic stroke treatment (Zhang et al., 2020). The inhibitory effect of ART on the proliferation of tumor cells is noticeable; however, a specific dose of ART is capable of promoting cell cycle progression in NSCs. Numerous studies have proven the protective effect of ART so that we can anticipate its therapeutic influence in the context of transplantation of NSCs after stroke. We will carry out further work in animal models and clinical trials to investigate and verify the direct effects of ART on NSCs after a stroke.

### Ginkgolide B

*Ginkgo biloba* extracts (GBE), a classic popular herbal product isolated from *Ginkgo biloba* L., has been reported to have neuroprotective and antioxidant effects in cognitive disorders and AD (Singh et al., 2019). Ginkgolide B (GB) is a terpene lactone component of GBE that shares its characteristics. GB exhibits beneficial antiosteoporotic effects on osteoblasts throughout the differentiation process *via* activation of the Wnt/β-catenin signaling pathway (Zhu et al., 2019). In a focal I/R model, IV administration of GB (20 and 40 mg/kg) has been demonstrated to cause marked reductions in infarct volumes, brain edema, and neurological deficits; furthermore, it inhibits I/R-induced NF-κB and microglia activation and proinflammatory cytokine production, which demonstrates the anti-inflammatory and antiapoptotic activities of GB (Gu et al., 2012). Due to the brain-protective effect of GB, Fang et al. (2010) suggested that the most appropriate therapeutic window for GB was within 4 h in a rat MCAO model; also, their data suggested that the BBB permeability of GB was increased after I/R given to its elevated concentration. Intraperitoneal injection of GB (20 mg/kg) decreased the neurological deficit score after MCAO (Zheng et al., 2018).

In addition to the neuroprotective effects of GB against CNS damage, it is essential to reveal the underlying mechanisms of its effects related to stem cell therapy. Zhu et al. (2019) found that 20 μM GB did not activate the proliferation of rat bone MSCs, however, the promotion of differentiation was positively correlated with the dose of GB. Research has also demonstrated that culturing normal mouse ESCs with GB induces cell apoptosis by influencing reactive oxygen species generation, activating JNK and caspase-3, decreasing mitochondrial membrane potential (MMP), and inhibiting cell proliferation. Furthermore, treatment with 10 mM GB causes the resorption of blastocysts after implantation and leads to decreased fetal weight (Chan, 2006). On the other hand, GB (20 nM) inhibits the proliferation of mouse NSCs derived from the postnatal subventricular zone but promotes NSCs differentiation into neurons through the Wnt/β-catenin pathway (Li et al., 2018). In another study, an NSCs cell line was treated with 40 and 60 mg/L GB, and a shortened cell growth cycle was observed. Mice treated with GB (20 mg/kg) once daily after MCAO exhibited increased proportions of nestin-positive, NSE-positive, GFAP-positive, and BrdU-positive cells, and the mRNA expression of BDNF and epidermal growth factor indicated that GB promoted the proliferation and differentiation of NSCs after MCAO (Zheng et al., 2018).

## Summary

Currently, the TCMs research literature includes only a small number of high-quality studies, most of which are necessary studies, and a few clinical articles. However, TCMs could be effective tools to promote the neuroprotection, antiapoptotic differentiation, and proliferation of NSCs after stroke and their clinical safety has been validated by years of study. Different doses of TCMs can lead to opposing effects that may be caused by natural shortcomings such as water insolubility and low bioavailability. Nose-to-brain drug delivery (NBDD) vastly improves the efficacy of TCMs treatments by increasing their bioavailability and absorption rates and avoiding drug-induced gastrointestinal tract irritation. Additionally, biocompatible organic substance formulations and novel metal and oxide nanoparticles can change medication profiles and reduce the required dose and side effects.

Classical pathways are involved in the effects of TCMs on the proliferation and differentiation of NSCs, including the Notch, PI3K/Akt, Wnt/β-catenin, and MAPK pathways. Molecules, including inflammatory factors, apoptosis factors, BDNF, and NGF are also involved. Studies have proven that almost all of these pathways/molecules mediate the process of CNS damage or support brain homeostasis maintenance. Ginsenoside Rg1 could promote the proliferation, differentiation, and maturity of NSCs through ERK1/2, Hes1 PI3K-AKT pathway, and p38 and JNK2 phosphorylation were under control after the admission of it in stroke models. Notch pathway was involved in AST induced differentiation of NSCs, NGF, IL-7, and BDNF-TrkB pathway took part in proliferation and the neuronal differentiation of NSCs after stroke *in vivo*. ICA promoted the proliferation and neurosphere formation of mouse NSCs induced by ERK but did not affect the differentiation of NSCs. The effect of AST and curcumin on NSCs after stroke was related to the Wnt/β-catenin pathway. PI3K/AKT/GSK3β/β-catenin was involved in the improvement of proliferation and differentiation of Sal B induced NSCs. PI3K/Akt/FOXO-3a/p27/Kip1 signaling was involved in the effects of ART after OGD *in vitro* and MCAO *in vivo*. The effects of TCMs on NSCs are regulated by multiple overlapping and interrelated signaling pathways that jointly constitute a regulatory network affecting neural regeneration. This kind of complex situation is probably related to drastic changes in physiology after lesion development. The interactions of TCMs and signaling pathways regulating the proliferation and differentiation of NSCs need to be further elucidated to clarify the multitarget and multi-pathway mechanisms and the comprehensive regulatory effects.

Large-scale clinical trials have led to a transformation in the clinical use of TCMs. However, issues remain. First, the ability of TCMs to cross the BBB and especially the enrichment rates in NSC pools in the subependymal region after infarction need to be determined quantitatively using high-performance liquid chromatography (HPLC), mass spectrometry, gas chromatography-mass spectrometry (GC-MS), or liquid chromatography-mass spectrometry (LC-MS). Second, specific molecular targets need to be identified. We need to return to the cellular level of stem research to explore the molecular mechanisms of TCMs because of the complexity of their systemic effects and because their neuroprotective effects may originate from sources other than the CNS. Also, studies on TCMs treatment combined with exogenous stem cell implantation are needed to determine whether TCMs can improve the microenvironment of stem cell proliferation after infarction. Such studies may provide new ideas for stem cell transformation therapies.

## Author Contributions

JW, XZC, and XJL reviewed the databases and analyzed information on subjects. HF, LT, and FW designed this review and worked on the manuscript revision. JW wrote the draft and modified this article. JH revised this article and replied to the reviewers in the modification phase. All authors contributed to the article and approved the submitted version.

## Conflict of Interest

The authors declare that the research was conducted in the absence of any commercial or financial relationships that could be construed as a potential conflict of interest.

## Abbreviations

AD: Alzheimer’s disease
ART: artesunate
AST: astragaloside IV
BBB: blood-brain barrier
BDNF: brain-derived neurotrophic factor
CNS: central nervous system
ERK: extracellular regulated kinases
ESCs: embryonic stem cells
GB: ginkgolide B
I/R: ischemia/reperfusion
ICA: icariin
IL: interleukin
iPSCs: induced pluripotent stem cells
JNK: c-Jun N-terminal kinase
MAPK: mitogen-activated protein kinase
MCAO: middle cerebral artery occlusion
MSCs: mesenchymal stem cells
NGF: nerve growth factor
NSCs: neural stem cells
NSPCs: neural stem/progenitor cells
OGD: oxygen-glucose deprivation
PI3K: phosphatidylinositide 3 kinase
PNS: panax notoginseng saponins
PSCs: pluripotent stem cells
RES: resveratrol
Sal B: salvianolic acid B
TCMs: traditional Chinese medicine monomers
Wnt/β-catenin: wingless/integrated/β-catenin.
